# Selenium efficiency in protecting sperm quality and testicular parameters of South African indigenous Zulu rams exposed to heat stress

**DOI:** 10.1007/s11250-024-04207-4

**Published:** 2024-10-19

**Authors:** Khomotso P. M. Lekola, Mthobisi S. Ngcobo, Khoboso C. Lehloenya

**Affiliations:** Department of Agriculture, University of Zululand, Private Bag X1001, KwaDlangezwa, 3886 South Africa

**Keywords:** Selenium, Heat stress, Sperm quality, Zulu ram, Semen

## Abstract

The study investigated selenium’s (Se) efficiency in preserving South African Zulu rams’ sperm quality and testicular parameters when they were exposed to heat stress. Indigenous Zulu rams (20) between 2 and 5 years old were allocated into four groups, namely the Se, testicular heat stress (THS), selenium plus testicular heat stress (SeTHS), and control. Each group comprised five rams; the groups were balanced according to the rams’ body weight and scrotal circumference. The Se and SeTHS groups received sodium selenite orally bi-weekly for 5 months. To induce heat stress, testicular heat insulation bags were wrapped around the testes of the rams receiving the THS and SeTHS treatments for 49 days. Semen was collected from the rams weekly from the third month onward; the first two months were for Se & THS acclimatization. In addition, testicular measurements were taken bi-weekly. Analysis of variance was used to analyze the sperm quality data. Duncan’s multiple range test was used to compare the groups’ data for significant differences. The results showed that the Se-supplemented rams’ scrotal circumference was smaller (*p* < 0.05) compared with the other groups. The Se, SeTHS, and control groups demonstrated similar total sperm motility; in contrast, the THS and SeTHS groups recorded low and high total sperm motility, respectively, compared with other treatment groups (*p* < 0.05). The semen from the rams that received THS without Se displayed a significantly higher number of immotile sperm cells (*p* < 0.05) and poor sperm quality, including total and progressive motility, and kinematic parameters when compared with other treatments, suggesting that Se protects sperm against THS. We concluded that selenium protected some sperm parameters (TSM, PSM, MV, VCL, VSL) of THS- treated rams while others did not improve (RV, NSM, C, STR).

## Introduction

Small stock, including sheep and goats, play a vital role in global food and wool production in tropical and subtropical areas, most of which are greatly affected by harsh climatic conditions (Ngcobo et al. 2022). Sheep are a major source of revenue and the backbone of many South African rural communities (Cloete et al. 2014). South African indigenous sheep breeds, including Damara, Namaqua Afrikaner, Pedi, and Zulu, are adapted to the country’s arid environmental conditions (Molotsi et al. 2019). These indigenous sheep play a vital role in the livelihoods of people as a source of meat, milk, wool, hide, and manure (Kunene et al. 2014; Selepe 2018). Amongst these indigenous breeds, the Zulu sheep breed is indigenous to the KwaZulu-Natal (KZN) province of South Africa and is renowned for its adaptability, durability, and inexpensive upkeep (Fossey et al. 2007). However, the breed’s genetic potential and productivity are deteriorating because of inbreeding and crossbreeding with exotic breeds (Scholtz 2005). The ram is an important member of the flock in conservation strategies, as it contributes 50% toward the genetic potential of the flock (Serrano et al. 2021). Mainly, the ram’s sexual behavior and semen quality restricts its ability to procreate (Zaher et al. 2020).

Environmental elements, such as humidity, daylight hours, and ambient temperature have an impact on the quality of semen (Kabukçu et al. 2020). Therefore, changes in temperature and humidity may interfere with spermatogenesis and induce thermal discomfort (Zaher et al. 2020). Climate conditions in South Africa’s subtropical regions are harsh throughout the summer and extend to other seasons as a result of climate change; this can lower male fertility by affecting the quality of semen (Llamas-Luceño et al. 2020). Heat stress caused by high ambient temperatures suppresses testicular functions through oxidative damage by producing reacting oxygen species, affecting intracellular signal transduction (Ahsan et al. 2014). Spermatozoa are typically shielded from oxidative damage by antioxidants and antioxidant enzymes found in the seminal plasma (Shaliutina-Kolešová et al. 2018). However, spermatozoa are vulnerable to oxidative stress when there is poor intracellular antioxidant activity, which may result from the reacting oxygen species levels surpassing normal values (Martín-Hidalgo et al. 2020).

Much attention has been focused on selenium (Se) since this important trace element possesses antioxidant qualities that are vital for spermatogenesis and reproduction; in addition, it plays a role in successfully repairing cell damage caused by stress (Petrujkić et al. 2014). The testis requires Se to produce testosterone and for the growth and development of spermatozoa (Cannarella et al. 2021; Petrujkić et al. 2014). In addition, Se is essential during spermatogenesis and semen collection to fight oxidative stress (Ebeid 2009). Normally, animals obtain Se from the grass or feed they eat (Hemingway 2003); however, in the KZN area of South Africa, heavy rainfall has left the soils and plants lacking in the mineral (Shabalala et al. 2013). Therefore, the goal of this investigation was to determine if Se offers protection to the testicular and sperm quality parameters of indigenous South African Zulu rams subjected to heat stress.

## Materials and methods

### Study site

The study was conducted at the University of Zululand’s (UniZulu; KZN, South Africa) dairy farm; further analyses were done at the University’s Department of Agriculture laboratory. The University is situated ~ 30 km southwest of Richards Bay ( 28.75°S, 32,03°E) and lies 121 m above sea level with an annual rainfall of 1,400 mm (Mbatha and Shikwambana, 2022).

### Management of animals

The ethical clearance for the study was granted by UniZulu’s ethics committee (UZREC 1171110-030 PGD 2021/20). The study was conducted using 20 indigenous Zulu rams between the ages of 2 and 5 years. The rams were separated from the ewes for the duration of the study. The rams were allowed to graze on natural pastures and had free access to water. Soil analysis was conducted to determine the level of Se in the soil, which was found to be below 0.001 ppm. Furthermore, the soil appeared to be slightly acidic at a pH of 4.30; acidity is known to severely decrease soil levels of Se (Gupta and Gupta 2000). The pasture samples from the grazing camps were taken, dried, and analyzed for Se; there were no traceable Se levels in the vegetation samples.

### Experimental design

The experiment was conducted from summer to autumn (January–May). The rams were grouped according to their body weight and scrotal circumference (Table 1), and allocated into four treatment groups of five rams. The treatment groups were arranged as follows: the first group’s rams were given Se supplementation only; the second group’s rams were subjected to THS; the third group’s rams were subjected to a combination treatment of Se and THS (SeTHS); and the fourth group’s rams were the control and not treated with dietary Se or THS. The rams supplemented with Se only were regarded as the positive control group, and those that did not receive Se supplementation or THS were the study’s negative control; the Se and SeTHS groups were the treatment groups. The rams subjected to dietary Se and combined SeTHS treatments were given sodium selenite dissolved in distilled water orally at a dosage of 0.34 Se mg/kg of body weight (Mojapelo and Lehloenya 2019). Dietary Se was administered once fortnightly for 5 months. Heat stress was induced by wrapping a thermal testicular insulation bag around the scrotum of the rams. The insulation bags were made with a double inner layer of soft cotton fabric, a middle layer of cotton fiber, and finally, an outer layer of denim fabric. The bag was secured at the top of the rump with an adjustable belt (Rocha et al. 2015). The scrotal temperature was taken using a digital oral thermometer by inserting it into the bag from the scrotal neck and placing it in the median of the scrotum; the recorded temperatures were between 36 and 38 °C. Semen collection started after 2 months (March) following Se supplementation and induced heat was allowed to acclimatize with spermatogenesis timing. The rams only wore testicular insulation bags for 49 days (7 weeks) to cover the first spermatogenesis cycle; the insulation bags were removed for the 3 months of data collection to avoid permanent damage to the Sertoli cells and the scrotal tissue.

**Table 1:** Body weight and scrotal circumference results for the control and experimental groups

	Sample size (*n*)	Se	SeTHS	THS	Control	*p* value
Body weight (kg)	5	39.7 ± 1.7	39.6 ± 1.4	38.26 ± 1.3	40.56 ± 1.4	0.92
Scrotal circumference (cm)	5	29.16 ± 1.2	29 ± 1.4	28.5 ± 1.4	29.6 ± 1.4	0.93

Values are mean **±** SEM. Selenium; Se, selenium plus testicular heat stress; SeTHS, testicular heat stress. SEM, standard error of the mean

### Semen collection and procedures

Semen was collected directly into a 15 ml test tube using an electro-ejaculator (3–6 volts for 3 s, at 7-second intervals) weekly for 3 months (Chella et al. 2017). The semen samples were transported to the laboratory in a styrofoam box at a regulated temperature of 37 °C and analyzed immediately after collection. Scrotal circumference and testicular parameters were assessed bi-weekly using a tape measure for scrotal circumference and a Vernier without a caliber for the testicular parameters. The measurements taken for the testicular parameters were classified as the length for the right (TL Right) and left (TL Left) testes, and the testicular breadth for the right (TB Right) and left (TB Left) testes.

### Sperm motility and concentration evaluations

Sperm quality parameters were evaluated using a computer-assisted sperm analyzer (Sperm Class Analyzer^®^ CASA system, v.5.1; Microptic S.L., Barcelona, Spain). Briefly, 10 µl of semen was diluted in 200 µl of saline in an Eppendorf tube at 37 °C for swim-up analysis. The sperm motility and concentration were visualized using a FireWire digital camera (black and white fast rate A602f Basler camera) connected to the CASA system. In addition, a 10 µl drop of the diluted semen was gently pipetted onto a pre-warmed glass slide and covered with a cover slip, then examined using a phase-contrast microscope (Wirsam Scientific, Johannesburg, South Africa) and Ph1 at 10× magnification (Jumintono et al. 2021). The sperm progression was classified as total sperm motility (TSM), progressive sperm motility (PSM), and immotile sperm cell (IMS). Sperm velocity was classified as rapid (RV), medium (MV), and slow (SV) velocity. The sperm kinematic parameters were classified as curve speed (VCL), linear speed (VSL), straight index (STR), and oscillation index (WOB). To ensure accurate concentrations, the volume of semen collected from each ram was entered on the animal information page of the CASA system under macro parameters, and the concentration was expressed in million (m)/mL (Bhalothia et al. 2022).

### Sperm morphology and viability evaluations

Sperm morphology and viability were evaluated by pipetting 10 µl of the swim-up sperm onto a warm slide followed by 10 µl of eosin-nigrosine; a free slide was then used to pull the mixture across the slide to form a smear. The stained slides were left to dry overnight at room temperature. The evaluation of sperm morphology for normal and abnormal cells and viability for live and dead sperm cells was based on counting at least 100 sperm cells under 60× magnification using an Olympus microscope (Evident Corp., Tokyo, Japan). Abnormal morphology was examined by identifying head, midpiece, and tail defects. The dead sperm cells had a pinkish-to-purple appearance, while live sperm cells remained white (Lukusa and Lehloenya 2017). The sperm morphology parameters assessed were normal sperm morphology (NSM) and abnormal sperm morphology (ASM); in addition, the viability of live (VBL) and dead (VBD) sperm were assessed.

### Data analysis

All data collected for testicular parameters, and sperm quality parameters were analyzed using the general linear model of Statistical Analysis Software (SAS^®^, v.9.4; SAS Institute Inc., Cary, NC, USA). The sperm motility parameters, including the TSM, PSM, IMS, RV, MV, SV, normal sperm morphology (NSM), abnormal sperm morphology (ASM), VBL, and VBD, were assessed. In addition, the sperms’ kinematics, including the VCL, VSL, STR, and WOB), and the testicular parameters were analyzed as repeated measures, considering binomial distribution using the Prog GLIMMIX procedure. Analysis of variance (ANOVA) was used to separate the data means. The Duncan test was used to determine the mean differences and were considered significant when *p* < 0.05. Data are presented as the mean ± standard error of the mean (SEM).

## Results

Table 2 shows the data (mean ± SEM) of the sperm quality parameters of the indigenous Zulu ram groups after Se supplementation, induced THS, or SeTHS compared with the control. The results show that the THS group’s TSM was lower (*p* < 0.05) compared with those of the control, Se, and SeTHS groups. The PSM was significantly higher (*p* < 0.05) in the control group compared with the treatment groups; similar PSM results (*p* > 0.05) were observed in the Se and SeTHS groups. Additionally, the PSM for the THS group was significantly lower (*p* < 0.05) compared with the results of the Se and SeTHS treatment groups and the control. The number of IMS was significantly higher (*p* < 0.05) in the THS group compared with the control group; however, the control and THS groups’ IMS results were not significantly different from the Se and SeTHS groups’ results.

Furthermore, the sperm velocity results showed that the number of sperm cells moving at an MV was significantly higher (*p* < 0.05) for the SeTHS group compared with the control and Se and THS treatment groups. The number of sperm cells moving at an RV was significantly higher (*p* < 0.05) in the control group compared with all the treatment groups; however, the SeTHS group’s RV sperm number was significantly lower (*p* < 0.05) compared with the control and Se and THS groups.

The study further analyzed the kinematic parameters of the sperm and found that the VCL and VSL were significantly higher (*p* < 0.05) in the control group compared with the treatment groups (Se, THS, and SeTHS). However, the VCL and VSL of the THS group’s sperm were significantly lower (*p* < 0.05) compared with the other treatment groups (Se and SeTHS); no significant difference (*p* > 0.05) was observed between the Se and THS groups. In addition, no significant differences (*p* > 0.05) were observed for the STR and WOB kinematic sperm parameters for the control and treatment groups.

The study further evaluated sperm morphology, including the NSM and ASM, and viability, including the VBL and VBD. No significant differences (*p* > 0.05) were seen in the NSM of sperm cells between the control and Se groups. However, the control and Se groups’ NSM results were significantly higher (*p* < 0.05) compared with those of the SeTHS and THS groups. The percentage of ASM was significantly lower (*p* < 0.05) in the control and Se groups, but significantly higher (*p* < 0.05) in the SeTHS and THS groups. The percentage of VBL was significantly higher (*p* < 0.05) in the control compared with the treatment group, and decreased sequentially from the control to the Se, SeTHS, and THS groups. The THS group had a significantly lower percentage of VBL compared with the control and Se and SeTHS treatment groups. In addition, significant differences (*p* < 0.05) were observed in the VBD percentages between the control and the treatment groups, which decreased sequentially (*p* < 0.05) from the control to the Se, SeTHS, and THS treatment groups. There was no significant difference (*p* > 0.05) in sperm concentration between the control and Se groups, however, sperm concentration in both groups was significantly higher (*p* < 0.05) compared with the SeTHS and THS groups.

**Table 2:** Sperm quality and kinematic parameter results of the control and treatment groups

Sperm quality parameters	Treatments			
	Control (*n* = 5)	Se (= 5)	SeTHS (*n* = 5)	THS (*n* = 5)
TSM (%)	72.6 ± 4.5^a^	69.4 ± 4.7^a^	69.3 ± 6.7^a^	55.2 ± 6.7^c^
PSM (%)	53.3 ± 4.1^a^	46.2 ± 4,1^b^	46.6 ± 4.1^b^	38.7 ± 4.1^c^
IMS (%)	27.1 ± 4.1^b^	31.2 ± 4.2^ba^	33.1 ± 4.2^ba^	44.8 ± 4.2^a^
RV (%)	39.7 ± 3.7^a^	32.0 ± 3.7^b^	14.6 ± 3.7^c^	29.9 ± 3.7^b^
MV (%)	21 ± 3.9^b^	23 ± 3.8^b^	92 ± 3.5^a^	14 ± 3.6^c^
SL (%)	12.29 ± 1.4	14.11 ± 1.4	12.97 ± 1.4	10.87 ± 1.4
VCL (µm/s)	139.8 ± 5.8^a^	130.7 ± 5.8^b^	135.9 ± 5.8^b^	116.5 ± 5.8^c^
VSL (µm/s)	79.5 ± 5.1^a^	70.5 ± 5.2^b^	74.1 ± 5.1^b^	64.2 ± 5.1^c^
STR (%)	65.8 ± 3.2	67.5 ± 3.7	66.9 ± 3.2	64.3 ± 3.2
WOB (%)	67.2 ± 2.6	63 ± 2.7	66.7 ± 2.7	63.8 ± 2.7
NSM (%)	81 ± 2.4^a^	77.1 ± 2.5^a^	55.4 ± 2.7^b^	52 ± 2.7^b^
ASM (%)	19 ± 1.4^a^	23.8 ± 1.4^a^	44.6 ± 1.5^b^	42.7 ± 1.5^b^
VBL (%)	87.5 ± 2.9^a^	80.5 ± 2.9^b^	71.6 ± 3.2^c^	62.3 ± 3.2^d^
VBD (%)	13. 5 ± 1.4^a^	19.9.5 ± 1.4^b^	29.7 ± 1.5^c^	37.7 ± 1.5^d^
Conc (m/mL)	31.83 ± 1.4^a^	30.71 ± 1.6^a^	26. 42 ± 1.4^ba^	21.34 ± 1.5^b^

Values are mean **±** SEM. Values with different superscripts within a row differ significantly (*p* < 0.05). TSM, total sperm motility; PSM, progressive sperm motility; IMS, immotile sperm cells; RV rapid velocity sperm cells; MV, medium velocity sperm cells; SL, slow; VCL, curve speed; VSL, linear speed; STR, straight index; WOB, oscillation index; NSM, normal sperm morphology; ASM, abnormal sperm morphology; VBL, viability live sperm cells; VBD, viability dead sperm cells; Conc, sperm cell concentration. Selenium; Se, selenium plus testicular heat stress; SeTHS, testicular heat stress; THS. SEM, standard error of the mean

Table 3 reports the scrotal circumference and testicular characteristics results of the Zulu ram control and treatment groups. The scrotal circumference average of the Se-supplemented rams was significantly lower (*p* < 0.05) compared with the control, THS, and SeTHS groups. No significant differences (*p* > 0.05) were observed in the rams’ testicular parameters between the control and Se, THS, and SeTHS treatment groups.

**Table 3:** Results of scrotal and testicular measurements of the control and treatment groups’ Zulu rams

Treatments	Scrotal and testicular parameters				
	SC	TL left	TL right	TB left	TB right
Control	29.17 ± 0.48^b^	11.34 ± 0.18	11.27 ± 0.23	5.67 ± 0.13	5.67 ± 0.14
(Se)	24.94 ± 0.40^a^	11.068 ± 0.21	11.03 ± 0.25	5.44 ± 0.13	5.35 ± 0.11
SeTHs	29.59 ± 0.47^b^	11.620 ± 0.17	11.53 ± 0.15	5.44 ± 0.12	5.54 ± 0.12
(THS)	29.98 ± 0.28^b^	11.52 ± 0.19	11.75 ± 0.20	5.59 ± 0.12	5.56 ± 0.12

Results are mean ± SEM; Se; selenium, SeTHs; selenium plus testicular heat stress, THS; testicular heat stress, SC; scrotal circumference; TL Left; testicular length left; TL Right, testicular length right; TB Left, testicular breadth left; TB Right, testicular breadth right (TB Right)

## Discussion

The presence of adequate Se in the male reproductive system is paramount for normal spermatogenesis and has a fundamental role in sperm maturation, thereby increasing the chances of conception (Ahsan et al. 2014). Sperm motility is a key trait in sexual reproduction that can be used to predict fertility. In the current study sperm motility parameters were used to determine the effect of Se on sperm quality of THS-treated Zulu rams. This study’s results show that the TSM in the control group rams was comparable to the group that received Se supplementation. The Se and SeTHS treatment groups showed increased PSM, while the THS-treated rams demonstrated reduced PSM; this suggests that Se protected sperm progression against induced THS. However, the control group had an even better PSM than the SE-treated rams, which is not in agreement with the findings of Lukusa et al. (2021). These authors found that Se supplementation improved rams’ sperm motility parameters.

Moreover, for spermatozoa to successfully pass through the female reproductive tract depends on rapid PM (Vasan 2011). Selenium supplementation reduced the sperms’ RV in the SeTHS group and did not improve the RV in the Se-only group. These results corroborate those of Piagentini et al. (2017), who found no direct effect of Se on sperm motility parameters. However, Se supplementation increased the MV of the SeTHS group’s sperm, highlighting that, although Se supplementation resulted in a satisfactory PSM, the RV of the SeTHS group’s sperm was compromised. This finding was supported when the sperm progression was further classified according to the sperm velocities, namely RV, MV, and SV. Furthermore, these results suggest that Se protects sperm cells by reducing their energy metabolism while reserving sufficient energy for the process of capacitation in the female reproductive tracts and providing better sperm endurance by allowing them to survive longer (Amaral 2022).

The over-production of reactive oxygen species and the animal’s exposure to heat stress result in detrimental effects, including changes in testicular structure and weight and lower-quality sperm cells (Hosny et al. 2020). There were many IMS cells in the semen collected from the rams that had been subjected to THS, indicating that heat stress had a detrimental effect on sperm quality. Sperm cells rely on their flagella beating to effectively maximize swimming efficiency through the viscous environment of the female reproductive tract to the ultimate goal of fertilizing the eggs (Guasto et al. 2020). Consequently, the kinematic analysis of sperm flagellar motility was essential in this study to understand the importance of the sperm cells’ speed and movement to fertilization. The kinematic results for the sperms’ VCL and VSL were compromised in the THS group but not the Se and SeTHS treatment groups. The results from the SeTHS rams suggest that Se has a protective effect and improves the vigorous and linear path of sperm cells in semen.

Moreover, the study’s results indicate that the NSM and ASM are affected by THS but not by Se supplementation. Additionally, the sperms’ VBL increase in the Se-supplemented group and was negatively affected by THS; however, the sperms’ VBL appeared to be protected by Se supplementation in the SeTHS group. Although Se did not reduce the number of VBD in the sperm of the Se-supplemented group, the number was reduced when Se supplementation was combined with THS treatment. In addition, Se supplementation increased the sperm concentration for the SeTHS group, while that of the THS group was negatively affected; however, these results were not significant and may signify that Se protects against induced stressors. Notably, the Se-supplemented group without THS showed a reduced scrotal diameter, which contradicts the results of Qazi et al. (2019), who found that Se supplementation increased the testicular size of male animals. This study’s results also illustrated that Se supplementation of THS-treated rams did not affect their testicular characteristics, including testicular length and breadth.

Furthermore, Se supplementation did not improve some sperm quality parameters, including the VBD, ASM, and RV, while improving others, such as the TSM, PSM, VCL, and VSL. The control group had satisfactory sperm quality parameters compared with the treatment groups; this may be because the basal level of Se supplementation used in the study is inadequate for indigenous Zulu rams; this is supported by Cannarella et al. (2021) who observed changes in sperm parameters when different Se concentration levels were administered. However, studies to evaluate Se efficiency at different levels of supplementation have shown that supplementation above the basal level can cause selenosis in animals (Zwolak and Zaporowska 2012). The study’s results also suggest that Se supplementation protects sperm cells against THS, given the similarities between the results of Se and SeTHS treatment groups.

This study faced several limitations, including that rams were not confined to a controlled temperature environment. Instead, they were allowed to graze freely in open fields, which could have exposed all the rams to hot climatic conditions. The Zulu sheep is a scarce breed, and its numbers are very low; therefore, only a low number of rams were included in the study, five per group. Serum Se levels were no examined since Se isotopes (organic and inorganic) are metabolized into hydrogen selenide which is then synthesized into various selenoproteins. Additionally, Hydrogen selenide can be methylated and excreted through breath and urine (Qazi et al. 2019). Prolonged Se supplementation of the rams was required for repeated semen collections. Based on the study’s findings, we concluded that scrotal insulation compromised the sperm quality parameters of rams, indicating that spermatogenesis and sperm maturation were altered. The results of the study further indicated that Se protected some of the THS rams’ sperm parameters (TSM, PSM, MV, VCL, VSL, VBL & VBD) while other parameters (RV, NM, C, IM, SL, ASM) did not improve. One mechanism of Se protection was the reduction in energy metabolism, which was demonstrated by the large number of MV sperm cells in the SeTHS rams’ semen. The study’s results also suggested that the effects of Se supplementation are enhanced when a stressor is encountered; this is supported by the similarities in the sperm quality parameters in the control and Se-supplemented groups only. Zulu sheep are indigenous to the hot-humid areas of the KZN province of South Africa. This breed is known and farmed for its disease resistance and adaptability to harsh environments, such as those of the coastal regions of the KZN province. What is still unknown is if the Zulu sheep only respond to foreign stressors, such as the THS treatment used in this study, and not to natural or environmental stressors, which requires further research.

## Data Availability

Data will be made available on request.
